# UnCanny: Exploiting Reversed Edge Detection as a Basis for Object Tracking in Video

**DOI:** 10.3390/jimaging7050077

**Published:** 2021-04-23

**Authors:** Wesley T. Honeycutt, Eli S. Bridge

**Affiliations:** Oklahoma Biological Survey, University of Oklahoma, Norman, OK 73019, USA; ebridge@ou.edu

**Keywords:** edge detection, object detection, video processing

## Abstract

Few object detection methods exist which can resolve small objects (<20 pixels) from complex static backgrounds without significant computational expense. A framework capable of meeting these needs which reverses the steps in classic edge detection methods using the Canny filter for edge detection is presented here. Sample images taken from sequential frames of video footage were processed by subtraction, thresholding, Sobel edge detection, Gaussian blurring, and Zhang–Suen edge thinning to identify objects which have moved between the two frames. The results of this method show distinct contours applicable to object tracking algorithms with minimal “false positive” noise. This framework may be used with other edge detection methods to produce robust, low-overhead object tracking methods.

## 1. Introduction

As image capturing hardware and storage capabilities improve, more and more digital imagery is being captured as video. Classic methods of edge detection based on first and second-derivative kernel operations, such as the Roberts [1], Sobel [2,3], Marr-Hildreth [4], and Haralick [5] techniques are still frequently used as the basis for modern static image edge detection due to the speed and quality of the output [6]. Perhaps one of the the most well-known and actively used techniques in the field of edge detection in computer vision is the Canny edge detector [7]. Briefly, this classic method detects edges by taking a de-noised grayscale image, finding the gradient intensities, suppressing spurious non-maxima, finding the dual-threshold of the result, and edge tracking by hysteresis. While the Canny technique has been improved many times over the years, including improving the blurring operator [8], using Otsu’s method for thresholding [9], and using advanced thinning techniques [10], most improvements on the Canny filter have focused on static images as opposed to video streams. Currently, many optical flow, object tracking, and motion detection algorithms are concerned with corner matching methods [11] that tend to require intensive computation [12], customized deep learning algorithms [13], and/or dedicated hardware [14]. As an alternative we offer a naive approach suitable for detecting moving objects that employs computationally inexpensive methods based on a reversal of classic edge detection techniques.

## 2. Methods

In this communication, we demonstrate edge detection reversal using the general steps from a single edge detection scheme, the Canny edge detector, and compare the results to simple object detection by image difference. Sample videos consisted of “moonwatching” footage wherein nocturnally migrating birds were filmed crossing in front of the moon as viewed through a spotting scope [15]. In an effort to present examples which are accessible to users with a background in applications, rather than exclusively those in computer science, all methods described here use functions available from the standard OpenCV and OpenCV-contrib libraries [16] in multiple programming languages.

### UnCanny Filter

The novel method, which we have dubbed the UnCanny filter, attempts to find differences in temporally adjacent images by applying the steps used in the Canny filter for edge detection in reverse order across two frames. The order reversal from the Canny filter suppresses spurious non-maxima prior to the execution of gradient intensities, and thereby reduces the impact of background noise prior to gradient operations arising from atmospheric effects or compression artifacts. The difference in the current frame of a video ($M_{n}$) and the previous frame ($M_{n-1}$) are passed through OpenCV’s adaptiveThresh with Gaussian filtering to produce a blurred image ($G({M^{\prime }}_{x},{M^{\prime }}_{y})$) which then is binarized by the Niblack method ($M^{\prime \prime }$) [17],

(1) $$M^{\prime }=M_{n}-M_{n-1}$$

(2) $$M^{\prime \prime }=G\left({M^{\prime }}_{x},{M^{\prime }}_{y}\right)-\bar{G({M^{\prime }}_{x},{M^{\prime }}_{y})}+255$$

The result is separated by anisotropic Sobel kernel operations in the horizontal and vertical directions, and each of these Sobel outputs is squared and added prior to taking the square root of the result:

(3) $$\begin{array}{cc} S_{x}=\left[\begin{array}{ccc} 1 & 0 & -1\\ 2 & 0 & -2\\ 1 & 0 & -1 \end{array}\right]\times M^{\prime \prime }, & \;S_{y}=\left[\begin{array}{ccc} 1 & 2 & 1\\ 0 & 0 & 0\\ -1 & -2 & -1 \end{array}\right]\times M^{\prime \prime }\\ S^{\prime } & =\sqrt{S_{x}^{2}+S_{y}^{2}} \end{array}$$

The scaled matrix was blurred again to reduce noise and simplified by the Zhang–Suen thinning algorithm (represented here as an operator (*Z*)) [18].

(4) $$U=Z\left(GS^{\prime }\right)$$

The output, already binary from the thinning step, may undergo contour detection steps. It should be noted that both the Sobel operations and linear distance steps shift the edges of the thinned contour away from the original object’s location; therefore, objects near the edge of the image may appear shifted after these detection steps.

## 3. Results and Discussion

### 3.1. Stepwise UnCanny Application to Video Frames

**Figure 1:**
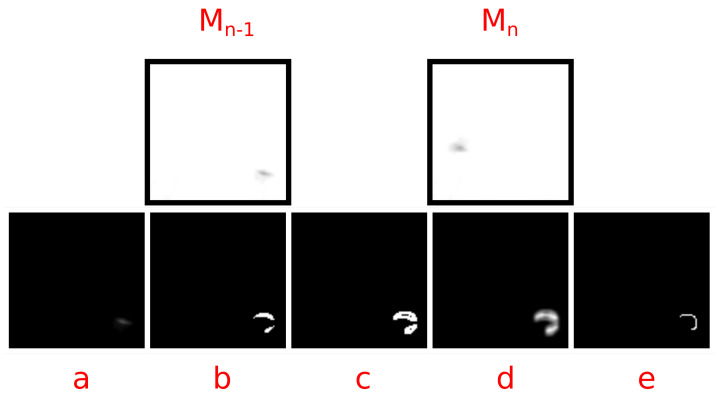
This image demonstrates a stepwise operation of the UnCanny filter comparing the current frame $M_{n}$ to the previous frame $M_{n-1}$. The images were (**a**) subtracted, (**b**) thresholded and blurred, (**c**) distanced after Sobel operation, (**d**) blurred, and (**e**) thinned.

By applying the method to temporally adjacent frames from a video, one can identify moving objects with a high “hit” rate. As an example, two adjacent frames from a video of migrating bird silhouettes passing in front of the moon, cropped to 100 px${}^{2}$, were processed using the UnCanny object detector; see Figure 1. Subtraction (Equation (1)) of the input matrices $M_{n-1}$ and $M_{n}$ yielded a difficult to see spot in Figure 1a. The difference image was processed with a Gaussian blur filter and thresholded (Equation (2)) to produce a binary image in Figure 1b. Edges of this blurred image were detected using the Sobel operation from Equation (3) in Figure 1c. The image was again blurred for Figure 1d and skeletonized for Figure 1e per the operations in Equation (4), producing visually distinct results.

The example images in Figure 1 were chosen and cropped to simplify the example for demonstrative purposes. Most edge detection methods would have little trouble detecting an object based solely on the difference between the input images represented by Figure 1a. However, object movement against visually complex backgrounds showcases the strengths of the UnCanny filter. In the case of the example video chosen for this demonstration, the lunar background is considerably unstable compared to nearer, less complex backgrounds. While the motion of the moon from frame-to-frame in a video may be negligible, especially if the image is artificially stabilized, the atmospheric lensing effects are not. Localized turbulent changes in the atmospheric pressure and relative humidity cause minute shifts in pixel illumination, especially in areas of sharp gradient contrast, such as near the lunar mare in the example image, a phenomenon known as atmospheric scintillation [19,20]. The result of these changes is that simple image differences in what appears to be a stable background become noisy to the point that the degree of blurring required to negate this noise would obscure the moving object. In the resolution of very long-distance imagery, which may be an edge-case example, the presence of atmospheric scintillation approximates compression artifacts in some video.

### 3.2. Comparison to Raw Frame Differences

**Figure 2:**
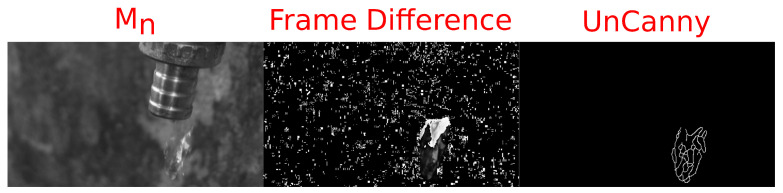
A wideo of water drops leaking from a faucet was processed with both a raw framewise difference and the UnCanny method. The leftmost image labeled $M_{n}$ shows the most recent frame of the video, using an 8-bit single-channel output. The middle image labeled “Frame Difference” visually depicts the results of a framewise subtraction and scaling of frames $M_{n}$ and $M_{n-1}$. The rightmost image is the output of the UnCanny method. In all images, a single droplet was seen falling from the faucet; however, the UnCanny method results shows only the movement of the drop, without shot noise.

The UnCanny filter results in a much lower output of false positives, defined here as false identifications of moving objects which do not correspond to any moving action in the frame, compared with the raw framewise difference. Stills from the application of both framewise subtraction ($M_{n}-M_{n-1}$) and the UnCanny method to single-channel video of water dripping from a faucet are shown in Figure 2. The public domain stock footage used here was encoded as 480×272 H.264 video in yuv420p color space at a bitrate of 949 kbits^−1^, a compressed format [21]. The water drop in this video is larger than the small objects detected in the previous example, demonstrating the UnCanny method’s capability extends beyond the extremely small. While the background in this video was stable and subject to constant lighting, by running a simple pixel difference across frames of the video, a large amount of shot noise was revealed. This simple image differencing approach is unacceptable for moving object detection. Treatment of the same frames with the UnCanny method results in a distinct droplet contour and no visual noise or false positives from the static portions of the frame.

**Figure 3:**
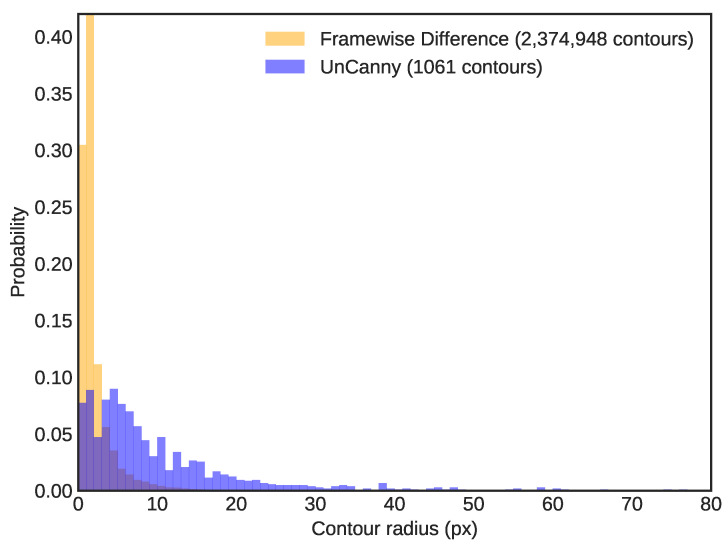
This is a normalized histogram of contour radii detected in the dripping faucet video. Bins were set at 1 px width. The simple framewise difference method reported 2,374,948 contours, while the UnCanny method reported 1061 contours over 34.46 s of input video. The proportion skews toward larger radii in the UnCanny filter, likely due to the high levels of visual noise or false positives in the simple framewise difference diluting “hits”.

While it is obvious that a simple image difference is unacceptable for moving object detection, it illustrates the gulf between rudimentary approaches and the UnCanny method. By analyzing the entirety of the sample video (34.46 s duration) and recording the sizes of all detected contours by minimum bounding radius for both the framewise difference and UnCanny methods, we illustrate the increase in object detectability. Figure 3 depicts a normalized histogram of contour radii with a bin size of 1 px. Application of the UnCanny method reduces the number of contours detected in the video by more than 2200×. Such a large reduction in results provides performance improvements to downstream calculations. Moreover, since most of the contour results ignored by the UnCanny method were due to shot noise, the radial size proportion in the results shift to higher values, which are more likely to correspond to positive moving object detection “hits”. While the framewise difference likely detected these same “hits”, these positive results were diluted by the extreme number of false positives.

### 3.3. Edge Detection Necessity

**Figure 4:**
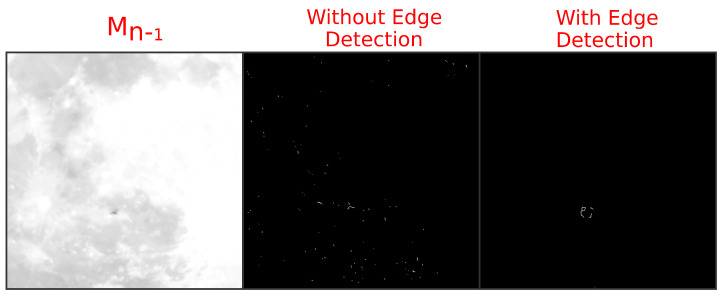
Performing edge detection is necessary for object detection on a complicated background. The image of the bird silhouette in front of the moon in the original image has been processed through the UnCanny detector without and with the Sobel edge detection steps. Excluding edge detection from the process produces an image with many errors generated by slight differences in the background between input images $M_{n-1}$ and $M_{n}$.

The use of image blurring and contour thinning techniques minimizes the impacts of background noise from the input images, but it cannot do so without edge detection as well. A 590.08 s section of compressed H.264 video in yuv420p color space with a bitrate of 884 kbits^−1^ of a nocturnal bird migration was cropped to a 500 px${}^{2}$ region of interest from 1920×1080 input. Figure 4 demonstrates this necessity by showing the results of the novel UnCanny object detector without including the edge detection step (Equation (3)) in a video frame of the previous bird silhouette further along its transit. While the complete UnCanny result shows a single area of white on the black background, indicating the previous position of the bird silhouette in frame $M_{n-1}$, the result without the benefit of edge detection included noisy speckles along the edges of the prominent lunar mare.

**Figure 5:**
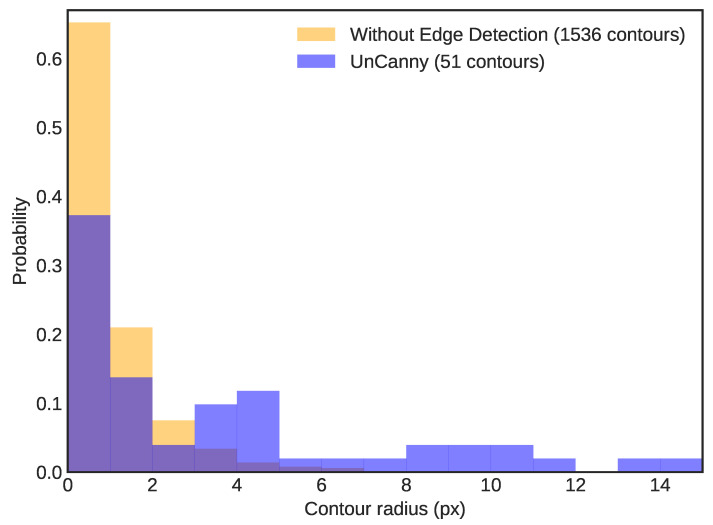
This is a normalized histogram of contour radii detected in the nocturnal bird migration video. Bins were set at 1 px width. Image processing including blurring, subtraction, and thinning steps, but excluding edge detection reported 1536 contours. The UnCanny method, which includes the edge detection step, reported 51 contours over 34.46 s of the same input video. The proportion skews toward larger radii in the UnCanny filter, likely due to the high levels of visual noise in the reduced method, diluting “hits”.

Similarly to the comparison with raw framewise difference in Section 3.2, the distribution of contour radii in the result images skews to larger contours when using the UnCanny method. A normalized histogram of contour radii with bins set at 1 px width (Figure 5) reports a significant reduction (30×) of unique contours in the UnCanny results compared to the same analysis without edge detection. The bulk of the extra contours have a bounding radius of <1 px, suggesting that they were false positives, smaller than the actual bird.

### 3.4. Limitations and Shortcomings

**Figure 6:**
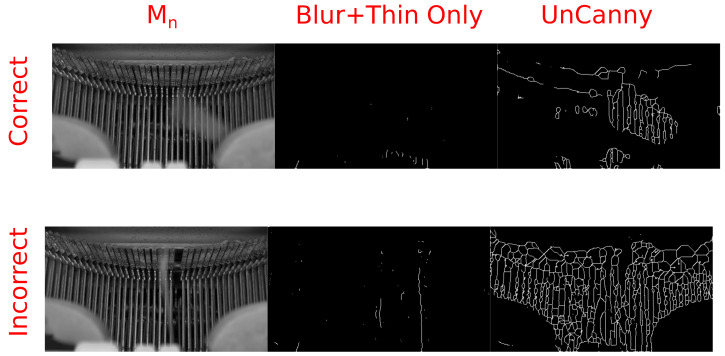
This figure shows examples of correct and incorrect motion identification by the UnCanny filter due to background vibration. In the correct example, the UnCanny method without edge detection (“Blur+Thin Only”) and with edge detection identify similar regions where the striker of the typewriter is moving. In the incorrect example, vibrations caused by the ribbon carrier progression (the blurred cylinders in the bottom of each input image) cause the UnCanny filter to report that the entire set of at-rest strikers is moving, rather than the single striker in motion near the center.

There are certain conditions that, when met, cause the UnCanny method to perform poorly. Video of a typewriter mechanism in Figure 6 demonstrates one instance of such a failure. In this H.264 video in yuv420p color space, out-of-frame keys are pressed on a mechanical typewriter, and video shows the mechanism of strikers hitting the platen as the ribbon carrier progresses [22]. During some frames, the ribbon carrier is still while the striker moves, generating a “hit”, identifying the moving region of the frame. In other frames, the ribbon carrier progresses the type guide, causing the UnCanny filter to identify this motion as well as the motion of the striker. When these events occur simultaneously the stationary portions of the typewriter vibrate slightly as the carrier moves, and the UnCanny filter erroneously reports that a large amount of the frame is in motion. This phenomenon is not observed using methods excluding edge detection.

**Figure 7:**
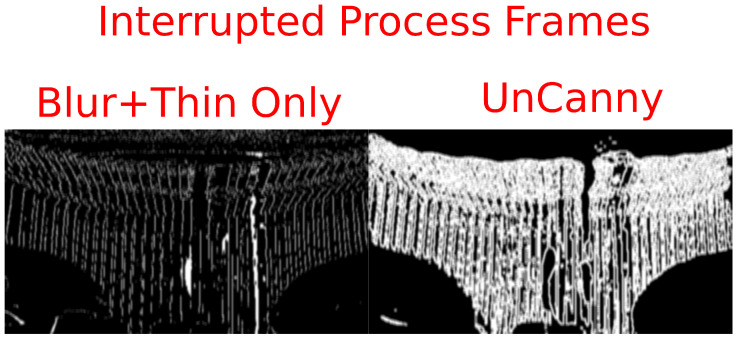
The cause of the previous incorrect detection by the UnCanny filter can be seen by observing an intermediate frame from the computer vision process. Depicted are the steps of the simplified “Blur+Thin Only” method and the UnCanny method prior to applying the edge thinning step. The Sobel edge detection step, only present in the UnCanny method, has made the strikers appear far more intense. This over-intensity due to minute vibrations was propagated to the final reported image.

This behavior occurs due to the enhancement of the edges during edge detection. By observing the output prior to the edge thinning step in both the simplified and UnCanny methods presented in Figure 7, the cause becomes clear. The Sobel operator here enhances the edges of the difference between frame $M_{n}$ and frame $M_{n-1}$, causing them to appear highlighted in Figure 7. In the method which does not include the Sobel operation, labeled “Blur+Thin Only” here, these minute vibrations of the background strikers are not as pronounced. Thus, when this intermediate frame is stage is processed by edge thinning, the entirety of the thick regions are included.

In practice, this means that the UnCanny method is suitable for complex but unmoving backgrounds. The steps reported in this demonstration of edge detection reversal do not include a robust background subtraction [23]. While several strong background subtraction algorithms exist within the OpenCV framework used for this work, including Gaussian Mixture Models (GMM) [24], improved GMMs [25], and Gale–Shapley matching [26], these were not included in the UnCanny filter. As the work presented here is intended as an application demonstration of edge detection to sequential video rather than as a report of a single technique, these methods were omitted for the sake of demonstrative simplicity.

### 3.5. Tracking Object Motion

**Figure 8:**
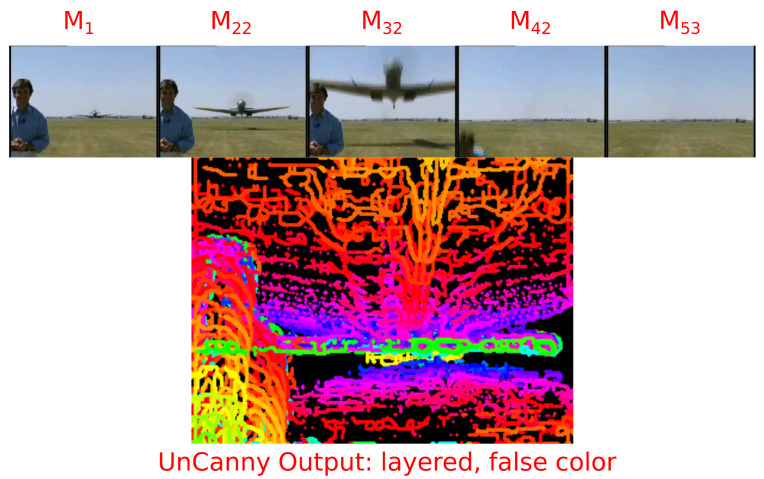
A video of a man ducking as a World War II era Spitfire warplane flies close overhead demonstrates the object tracking capabilities of the UnCanny method. A few frames of the video sequence are shown first to demonstrate the action and motion in the video. The colorful image below the action sequence reports the contours discovered by the UnCanny filter layered sequentially in color gradient. The action in the video generates distinct shapes related to the objects in motion, including the plane, the flinching man, the shadow of the plane, and the horizon as air currents from the plane disturb the camera.

Applying the UnCanny method to sequential frames of moving imagery allows for object tracking. In a sample video encoded in H.264 in yuv420p color space of a man flinching as a warplane flies above his head, we can illustrate these tracks [27]. Each frame was processed by the UnCanny filter, and the detected object contours were overlaid in sequential color. The result shown in Figure 8 depicts four moving regions. The first is the warplane approaching the man and flying overhead. The second is the outline of the man as he flinches during the flyover. The third is the shadow of the warplane on the grass. Finally, the fourth region is the horizon line which shifts slightly due to the vibration of the camera in the wake of the low-flying plane.

**Figure 9:**
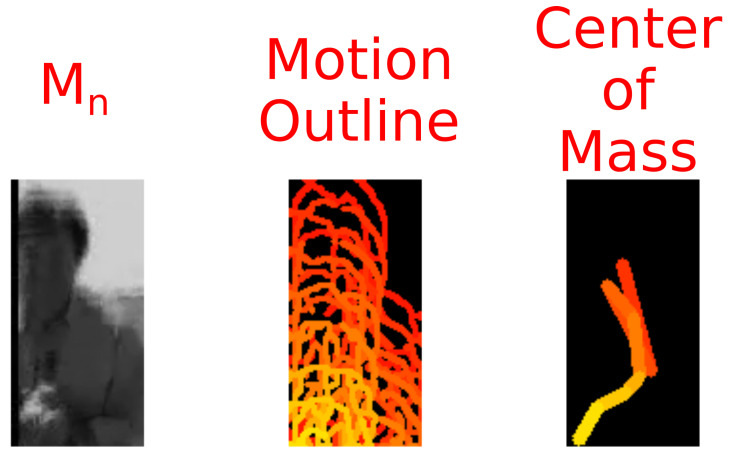
By focusing on regions of interest in the video of the Spitfire flyby, we can track the actions of individual components. Here, most of the frame has been cropped, leaving only the scared reporter flinching as the plane flies above. As mentioned in Figure 8, the UnCanny filter determines the motion as outlines of the actor. By calculating the center of mass of these outlines, we can determine the movement vector. In the rightmost image, the centroids from the center image are shown as connected lines, demonstrating this movement vector visually.

The individual regions of interest in this image may be tracked separately using the output from the UnCanny method. By isolating a region of the frames featuring the flinching man, we see this demonstrated in Figure 9. The rightmost portion of this figure shows the shift in center of mass of the largest contour on the cropped frame. The total vector of motion across these points trends downwards as the man flinches. Other techniques may be used at this point to determine motion more precisely, such as object pose matching [28,29] or partially supervised pattern recognition [30,31].

### 3.6. Computational Efficiency

To demonstrate the computational efficiency of the UnCanny filter, a comparison was run between it and another well-known method, Lucas–Kanade optical flow. Video processing ran on approximately three-million frames of video using hardware with an Intel Core i7-7500U 2.70 GHz “Kaby Lake” processor to yield relative computational time expenditure. Each object tracking method was run sequentially on a single processor thread, and frame acquisition time was not measured. For comparison, a simple differencing operation was executed which applied a Laplacian gradient to input frames and took the difference of the binarized result. Laplacian matrices used were of the same size as the Sobel matrices applied in the UnCanny filter The UnCanny filter used 1.48× computational time as the Laplacian gradient, while Lucas–Kanade optical flow used 8.06× computation time. While object detection methods and complex optical flow methods are not directly comparable from a theoretical standpoint, the time comparison against a frequently employed method serves to demonstrate the relative quickness of the UnCanny filter. It must be noted that computation time is not a reliable measure of process efficiency, quantity of output, or “usefulness”; and the efficiency calculated here purely demonstrates the reduced computational complexity of the UnCanny method compared with other well-known methods.

## 4. Conclusions

As video recording and storage capabilities increase, and as the demand for computer vision applications for these videos likewise increases, it is worth adapting classic edge-detection methods to address these advances. Our framework is capable of object detection with fewer false positives at minimal computational expense. By reversing the techniques from conceptually-simple edge detection methods, such as the Canny filter, it is possible to detect differences between sequential images with greater accuracy than raw subtraction. Although for the sake of brevity, this paper only demonstrated the reversal of the Canny filter, the higher-level concept of reversing edge detection for framewise analysis of video would benefit from further exploration. Many more edge detection methods for static images exist, and many more improvements have been made on each since their inception. Each of these holds potential for facile object detection with low computational overhead. Furthermore, none of the methods presented here require advanced computer programming to implement, because each step is possible within the user-accessible OpenCV code base. The initial application of this method was limited in scope, and the authors encourage computer vision experts to expand upon this framework in other contexts where moving-object detection is required under the constraints of low-cost video analysis.

## Data Availability

All sample images were taken from video data stored in an Open Science Framework repository: https://osf.io/52kyq/ or are public domain video from https://archive.org/ accessed on 7 April 2021.
